# Progestins alter photo-transduction cascade and circadian rhythm network in eyes of zebrafish (*Danio rerio*)

**DOI:** 10.1038/srep21559

**Published:** 2016-02-22

**Authors:** Yanbin Zhao, Karl Fent

**Affiliations:** 1 University of Applied Sciences and Arts Northwestern Switzerland, School of Life Sciences, Gründenstrasse 40, CH–4132 Muttenz, Switzerland; 2 Swiss Federal Institute of Technology (ETH Zürich), Institute of Biogeochemistry and Pollution Dynamics, Department of Environmental System Sciences, CH–8092 Zürich, Switzerland

## Abstract

Environmental progestins are implicated in endocrine disruption in vertebrates. Additional targets that may be affected in organisms are poorly known. Here we report that progesterone (P4) and drospirenone (DRS) interfere with the photo-transduction cascade and circadian rhythm network in the eyes of zebrafish. Breeding pairs of adult zebrafish were exposed to P4 and DRS for 21 days with different measured concentrations of 7–742 ng/L and 99-13´650 ng/L, respectively. Of totally 10 key photo-transduction cascade genes analyzed, transcriptional levels of most were significantly up-regulated, or normal down-regulation was attenuated. Similarly, for some circadian rhythm genes, dose-dependent transcriptional alterations were also observed in the totally 33 genes analyzed. Significant alterations occurred even at environmental relevant levels of 7 ng/L P4. Different patterns were observed for these transcriptional alterations, of which, the *nfil3* family displayed most significant changes. Furthermore, we demonstrate the importance of sampling time for the determination and interpretation of gene expression data, and put forward recommendations for sampling strategies to avoid false interpretations. Our results suggest that photo-transduction signals and circadian rhythm are potential targets for progestins. Further studies are required to assess alterations on the protein level, on physiology and behavior, as well as on implications in mammals.

Synthetic progestins find application in contraception and medical treatments. Progesterone (P4) and progestins are excreted as parent compounds and metabolites by humans and animals. Thus, P4 and its metabolites, as well as several synthetic progestins occur in wastewaters. These steroids enter aquatic ecosystems by wastewater but also by agricultural run-off^1^,^2^. In surface water, they occur in the low ng/L range^1^,^2^,^3^,^4^. Residues of P4 had been detected at concentrations of up to 30.5 ng/L in surface waters^5^, as well as several other progestins, which occur in the ng/L range^5^,^6^. Besides aquatic wildlife, humans may also be exposed unintentionally to progestins via contaminated drinking water and seafood^7^,^8^.

Adverse effects of environmental progestins on aquatic organisms have been studied only recently^1^,^2^. Based on their hormonal activities, potential impacts on the sex hormone system and on reproduction, including decreased fecundity were analyzed^9^. The hypothalamic-pituitary-gonadal (HPG) axis, hormone levels, histology of gonads and reproduction were found as the common targets, which were affected even at environmental relevant concentrations^9^,^10^,^11^,^12^,^13^,^14^.

Thus far, little attention has been paid on exploring other potential effects on additional biological endpoints. Toxicogenomic analyses of progestins showed that besides alterations of molecular signals involved in hormone homeostasis and reproductive process, several novel pathways were uncovered, such as circadian rhythm and cell cycle regulation^11^,^12^. Most prominent were transcriptional alterations of key genes involved in circadian rhythm signals, such as *nr1d1, per1b* and *cry5*, in addition to genes involved in the HPG-axis. They occurred even at 3.5 ng/L P4 and 4.8 ng/L dydrogesterone^11^,^14^. Recently, we reported a detailed analysis of the deregulation of circadian rhythm in zebrafish following P4 and DRS exposure^15^. We investigated 41 circadian rhythm genes (subdivided into 13 groups) for a comprehensive analysis of the circadian rhythm network. Thereby, we proved evidence that the circadian rhythm and related downstream pathways were disrupted by these progestins, most prominently in brain and liver, but not in gonads.

It is known that in addition to the brain, eyes are responsive to circadian oscillators. In our transcriptomics studies, we also noted that a group of biomarker genes involved in photoreception or against UV stress were significant altered by progestins, such as *opsin3*^12^ and *ddb2*^11^,^12^, suggesting potential effects on light response. In zebrafish, circadian rhythm genes in eyes display highest intensity of oscillations, as shown for the *clock* gene, followed by those in kidneys and brain^16^. We confirmed the interference of progestins with the circadian rhythm signals in the brain, but did not include eyes^15^. Thus, it is presently unknown, whether alteration of circadian rhythm observed in zebrafish brain is extended into eyes. Clinical reports suggested potential eye dysfunctions in humans can be caused by progestins, since side effects, such as swollen eyes, blurred vision, diplopia and other vision changes were reported for several progestins used in hormone therapy^17^,^18^. Therefore, the question arises, whether there are progestin-induced molecular effects in the eye, including alterations in photo-transduction cascade and circadian rhythm.

To test this hypothesis, we analyzed the transcriptional responses in eyes of zebrafish originating from our experimental exposure of zebrafish to P4 and DRS for 21 days^15^. Here we report effects in eyes as an extension to this study that had a different focus, such as reproductive and transcriptional effects in brain, liver and ovary. In the eyes, we conducted a comprehensive analysis of genes involved in the photo-transduction cascade and circadian rhythm network, and confirmed that progestins can affect the molecular regulation of photo-transduction and circadian rhythm.

## Results and Discussion

### Target genes

The focus of the present study lied on transcriptional alterations of 43 genes belonging to the photo-transduction cascade and circadian rhythm in zebrafish eyes. Our previous study with environmental progestins suggested alterations in the abundance of *opsin3* and *ddb2*^11^,^12^, and thus potential effects on the light response. Therefore, we evaluated a series of key genes involved in the photo-transduction cascade. In addition, we investigated whether alterations in the circadian rhythm signals in the brain^15^ was extended into eyes. Considering the tight interactions between photo-transduction and circadian rhythm signals^19^,^20^, in the present study we aimed to provide a comprehensive analysis into potential molecular effects of environmental progestins on zebrafish eyes.

Circadian rhythm genes and photo-transduction cascade genes both display remarkably circadian oscillations with a period of 24 h. Previous studies showed that for some of these genes, including *per1b* and *nr1d1*, marked changes in mRNA abundance occurred during a 24 h period, and significant changes even occurred within a few hours^20^,^21^. In our experiments, time differences between sampling of controls and progestin exposed zebrafish spanned up to 5 hours (CT1–CT6). Due to practical constraints, a shorter sampling time was not possible, with the implication that there might be false positive answers, when solvent controls and progestin exposed zebrafish were compared, although not sampled at the same time. Therefore, to avoid such an effect, we employed unexposed fish with the same strain, age and breeding conditions as negative control following our exposure experiment.

We demonstrated that there were no significant differences in transcription levels of all these genes in both females and males between unexposed (normal) fish and our solvent control fish in the exposure experiment (Fig. S1). Thus, the transcriptional levels can be compared at each time point after normalization, which would be crucial for evaluating their actual alterations in response to P4 and DRS. A detailed discussion of these effects follows below.

### Histology and transcriptional alterations of photo-transduction cascade genes

**Figure 1:**
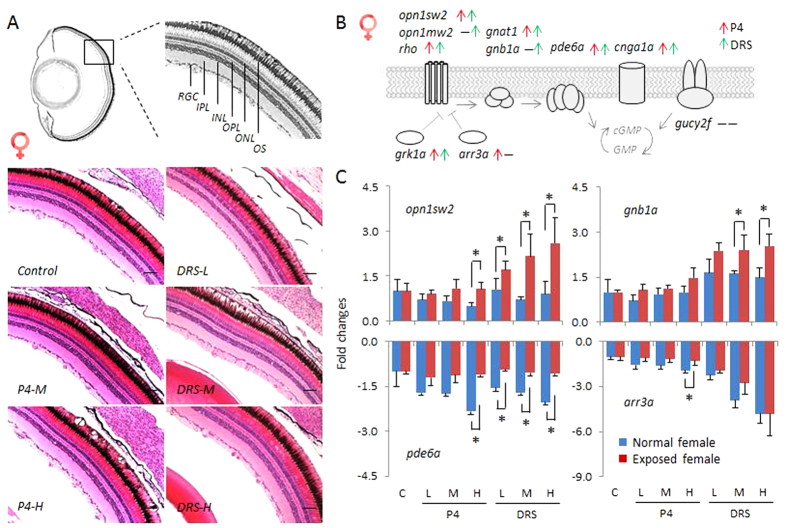
Histology and transcriptional responses of photo-transduction genes in eyes of females. (**A**) Transverse sections of eyes of female zebrafish exposed to solvent control, and different concentrations of P4 and DRS. Upper diagram represents the sampling section of zebrafish eye, lower diagram responses to P4 and DRS. Scale bar = 50 μm. Key: RGC: retinal ganglion cell; IPL: inner plexiform layer; INL: inner nuclear layer; OPL: outer plexiform layer; ONL: outer nucleus layer; OS: outer segment. (**B**) Schematic diagram depicts the photo-transduction cascade in zebrafish and ten key genes measured in the present study. Red arrow: up-regulated genes in response to P4. Green arrow: up-regulated genes in response to DRS. (**C**) Transcriptional responses of four key genes (*opn1sw2, gnb1a, pde6a* and *arr3a*) expressed as fold-changes compared to unexposed (normal) females sampled at the same time-point. Blue bars in each figure represent gene expressions of unexposed (normal) females. Red bars in each figure represent gene expressions of exposed females; Solvent control and P4 and DRS-exposed. Key for concentrations (red bars): P4: L: low dose (7 ng/L); M: middle dose (116 ng/L); H: high dose (742 ng/L). DRS: L: low dose (99 ng/L); M: middle dose (2´763 ng/L); H: high dose (13´650 ng/L).

**Figure 2:**
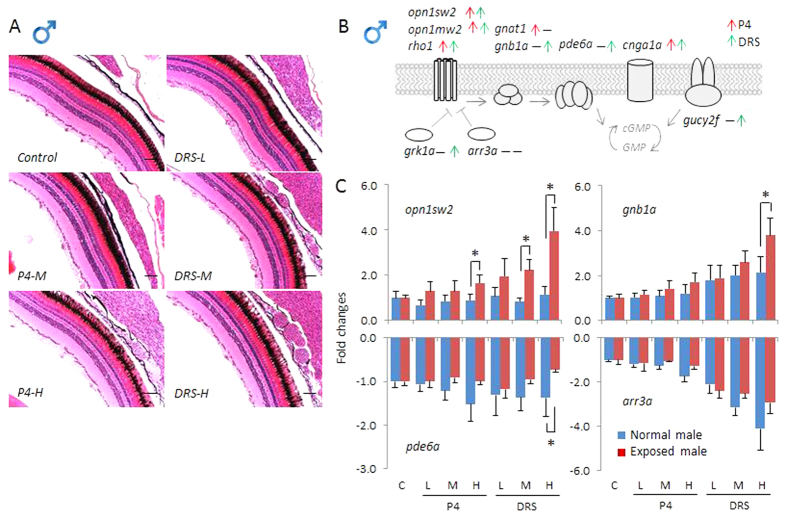
Histology and transcriptional responses of photo-transduction genes in eyes of males. (**A**) Transverse sections of eyes of male zebrafish exposed to solvent control, and different concentrations of P4 and DRS. Scale bar = 50 μm. (**B**) Schematic diagram depicts the photo-transduction cascade in zebrafish and ten key genes measured in the present study. Red arrow: up-regulated genes in response to P4. Green arrow: up-regulated genes in response to DRS. (**C**) Transcriptional responses of four key genes (*opn1sw2, gnb1a, pde6a* and *arr3a*) expressed as fold-changes compared to unexposed (normal) males sampled at the same time-point. Blue bars in each figure represent gene expressions of unexposed (normal) males. Red bars in each figure represent gene expressions of exposed females; Solvent control and P4 and DRS-exposed. Key for concentrations (red bars): P4: L: low dose (7 ng/L); M: middle dose (116 ng/L); H: high dose (742 ng/L). DRS: L: low dose (99 ng/L); M: middle dose (2´763 ng/L); H: high dose (13´650 ng/L).

First, we evaluated the morphological organizational architecture of adult zebrafish eyes exposed P4 and DRS. Though slight swelling of the eyes and exophthalmos were observed during the sampling at high P4 and DRS concentrations, we observed no significant morphological alterations. Detailed histological analysis demonstrated that the thickness of the retina, as well as the structures of different cell types, like the retinal ganglia cells and inner plexiform layer showed no significant alterations in both females and males (Figs 1A and 2A).

The molecular components of the photo-transduction cascade signal is known in vertebrates^22^,^23^. A similar cascade was established in zebrafish. It includes ten key genes, divided into seven different groups, as shown in Fig. 1B. The *opsin1* and *rho* families are light-sensitive proteins found in photoreceptor cells of the retina, which are the first step in the visual transduction cascade, crucial for the mediation of the conversion of photons of light into electrochemical signals. These signals are then transferred into the cell thought two other protein families, transducin (*gnat1*, *gnb1a*) and pde6 (*pde6a*). In addition, these genes are also regulated by several other genes, like rhodopsin kinase (*grk1a*), arrestin (*arr3a*), cyclic nucleotide-gated channel alpha 1 (*cnga1a*) and guanylyl cyclase (*gucy2f*)^22^,^23^.

We found more significantly up-regulated genes, as well as relative higher fold changes of transcripts in female than male zebrafish. Of these genes, seven and five, respectively, were significantly up-regulated in females and males exposed to P4, as compared to those in normal fish (Figs 1B, 2B and S2), which were sampled at the same time of the day. Similarly, in females and males exposed to DRS, transcripts of eight and seven genes, respectively, were significantly up-regulated (Figs 1B, 2B and S2). We found no gene transcript with significant down-regulation. Specifically, we observed several different patterns of transcriptional alterations. First, we found a remarkable up-regulation of some genes in response to high concentrations of P4 and DRS, like *opn1sw2*, while in unexposed (normal) fish, the abundance of transcripts remained stable during the entire sampling time (Figs 1C and 2C). Second, transcripts of several other genes, including *gnb1a* and *opn1mw2*, displayed an increase in unexposed (normal) fish during the sampling time, while much higher up-regulations were observed for the progestin-exposed zebrafish (Figs 1C, 2C and S2). Thus, normal time-related expressional changes were enhanced by progestins. Third, transcripts of some genes, such as *pde6a* and *arr3a*, that decreased in time in unexposed fish were significantly less down-regulated in response to high concentrations of P4 (*pde6a*, *arr3a*) and at all DRS concentrations (*pde6a*), especially for the females (Figs 1C and 2C). Thus, the down-regulation was attenuated.

These results indicate significant transcriptional increases of photo-transduction signals. For some transcripts, like *opn1sw2* and *gucy2f*, up-regulation even reached up to about four times. It should be noted that *arr3a* (Fig. 1C) and *grk1a* (Fig. S2), two genes involved in inhibition of photo-transduction signal, were significantly attenuated by progestins. This seems an effect of compensation, observed in other responses occurring in the HPG-axis in reaction to environmental chemicals^24^,^25^. In mammals, there are more than 30 genes belonging to the photo-transduction cascade^22^,^23^ and probably even more in zebrafish due to genome duplication. We performed homologous alignments in the zebrafish genome based on Ensembl database, and identified 59 genes in total, which can be subdivided into 10 groups (data not shown). To gain more detailed insights into regulation of the whole complex regulatory mechanism in eyes by progestins, further studies that include a higher number of genes should be performed.

### Transcriptional alterations of circadian rhythm

The basic molecular mechanism of the biological circadian clock in mammals consists of six groups of genes (*CLOCK*, *ARNTL*, *PER*, *CRY*, *NR1D* and *RORC*)^26^. These genes comprise the essential four feedback loops. Of these, *CLOCKs* and *ARNTLs* heterodimers form the core component. It activates the transcription of paralogs of *PERs* and *CRYs*, which are transcriptional repressors form the negative limb of the feedback loop that inhibit *CLOCKs*/*ARNTLs* heterodimer activity, and thereby negatively regulating their own expressions. It also activates the transcription of nuclear receptors, *NR1D1/2* and *RORs*, which form the second group of feedback loops that repress and activate the transcriptions of *CLOCK/ARNTL* heterodimer, respectively^26^. Besides these core genes, novel circadian rhythm genes were discovered, such as *NFIL3, DECs*, and *TEFs*^27^,^28^,^29^,^30^.

In zebrafish, the molecular mechanisms of circadian rhythm appear to have much in common with the mammalian system, while there are also some differences^31^. Due to multiple copies of *clock*, *per*, *cry*, *rorc* and other genes in zebrafish, a more complex pool of regulatory factors exist^19^. In our previous study, we developed a comprehensive circadian rhythm gene network for zebrafish, which combined 41 circadian genes subdivided into 13 groups^15^. By use of this pool, we systematically assessed the alterations of circadian signals on the molecular level and deciphered potential effects of P4 and DRS in the brain of zebrafish.

**Figure 3:**
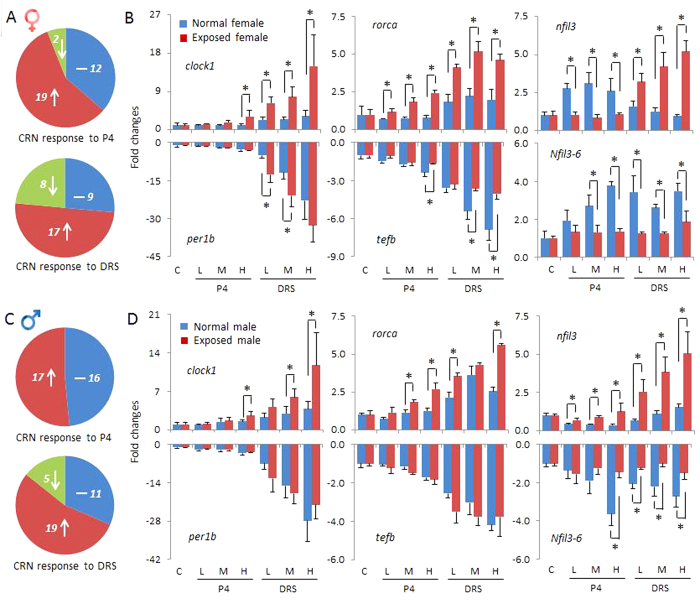
Transcriptional responses of circadian rhythm (CRN) genes in eyes of zebrafish. (**A**) Proportion of genes with altered transcriptional responses in females to various concentrations of P4 (upper) and DRS (lower) presented in colors and numbers of gene transcripts. Blue: numbers of genes without significant alterations. Red: numbers of up-regulated genes. Green: numbers of down-regulated genes. (**B**) Different types of transcriptional responses expressed as fold-changes compared to unexposed (normal) females sampled at the same time-point. Blue bars in each figure represent gene expressions of unexposed (normal) females. Red bars in each figure represent gene expressions of exposed females; C, solvent control and P4 and DRS-exposed. (**C**) Proportion of genes with altered transcriptional responses in males to various concentrations of P4 and DRS presented in colors and numbers of gene transcripts. (**D**) Different types of transcriptional responses expressed as fold-changes compared to the unexposed (normal) males sampled at the same time-point. Key for concentrations (red bars): P4: L: low dose (7 ng/L); M: middle dose (116 ng/L); H: high dose (742 ng/L). DRS: L: low dose (99 ng/L); M: middle dose (2´763 ng/L); H: high dose (13´650 ng/L).

**Figure 4:**
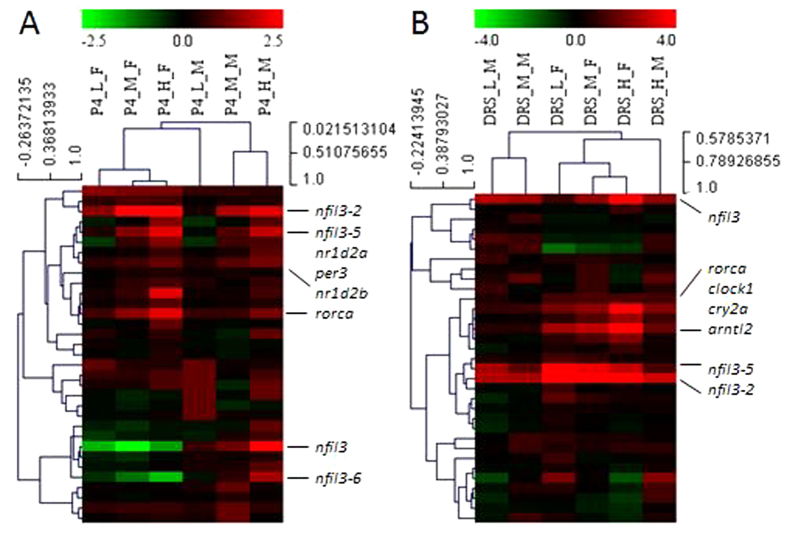
Hierarchical clustering depicts the whole transcriptional responses in eyes of females and males. (**A**) Female (_F) and male (_M) zebrafish eyes after exposure to different doses of P4. (**B**) Female (_F) and male (_M) zebrafish eyes after exposure to different doses of DRS. Key for concentrations: P4_L: low dose (7 ng/L); P4_M: middle dose (116 ng/L); P4_H: high dose (742 ng/L). DRS: L: low dose (99 ng/L); M: middle dose (2´763 ng/L); H: high dose (13´650 ng/L).

**Figure 5:**
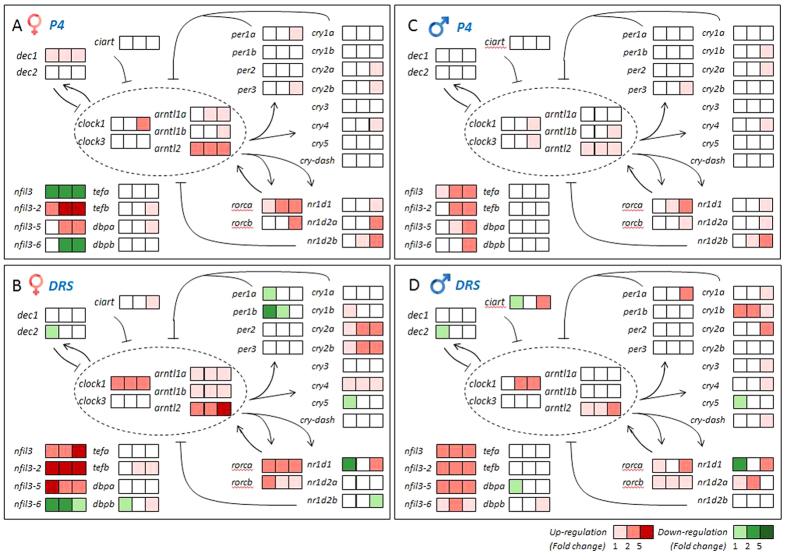
Heat map depicts the whole pattern of transcriptional changes in circadian rhythm network in eyes. (**A**,**B**) Heat map of eyes of females after exposure to different doses of P4 (**A**) and DRS (**B**) in a color scheme. (**C**,**D**) Heat map of eyes of males after exposure to different doses of P4 (**C**) and DRS (**D**) in a color scheme. Gene expressions are expressed as fold-changes compared to unexposed (normal) fish sampled at the same time-point and displayed in colors. Horizontal row of squares represent three doses from low, middle to high (from left to right). The legend listed in the lower right corner of the graph gives the thresholds for different colors. Numbers (1,2,5) mean boundaries between each color, representing fold-changes. Light red: fold changes between 1–2-times; Middle red: fold changes between 2–5-times; Dark red: fold changes higher than 5-times, and similarly for the down-regulation in green colors. Gray represents no significant alterations.

In the present study, we employed the same circadian rhythm gene network to explore potential effects of P4 and DRS on zebrafish eyes. In total, we analyzed 33 circadian genes, which were found to display significant alterations in response to various doses of P4 and/or DRS in the zebrafish brain^15^. P4 significantly altered the circadian network in eyes of both females and males. Of these circadian genes, 19 were significantly up-regulated and two down-regulated at different P4 concentrations in females (Fig. 3A). In males, 17 genes were significant up-regulated, while no genes displayed significant down-regulations (Fig. 3C). The hierarchical clustering and heat map also revealed these two subdivisions of genes with up-regulations and down-regulations, respectively (Figs 4A and 5A,C). The transcriptional alterations for some genes, like *arntl2* and *nfil3*, even occurred at environmental relevant concentrations of 7 ng/L P4 (Fig. 5A,C).

Compared to P4, DRS displayed a quite similar, but not identical, pattern of transcriptional responses (Figs 3, 4B and 5B,D). First, transcriptional alterations were more pronounced, as indicated by the higher number of significant alterations observed at lowest DRS dose (Fig. 5B,D). Second, a group of circadian genes, including *per1b* and *nr1d1*, showed down-regulations in females (Fig. 5B) and males (Fig. 5D), while this was not observed for P4 (Fig. 5A,C).

Detailed analysis further revealed the different patterns among these transcriptional responses (Fig. 3B,D). For some genes, slight but significant transcriptional increases were observed for unexposed (normal) females during the sampling time span, while they displayed much higher up-regulations to high concentrations of P4 and DRS. This enhancement was observed for *clock1* and *rorca* transcripts. Several transcripts that decreased in abundance in time in unexposed females were remarkably attenuated in response to high concentrations of P4 and DRS, like *tefb* in females (Fig. 3B) and less in pronounced in males (Fig. 3D). Alterations of the *nfil3* family transcript were most significant with highest alterations and multiple patterns of responses (Fig. 3B,D). Of them, *nfil3-2* and *nfil3-5* displayed most significant alterations in both genders of up to 5–6 times fold, similar to *clock1*. *Nfil3* and *nfil3–6* displayed a quite different response pattern than other genes (Fig. 3B,D). Genes of *nifl3* family are basic leucine zipper transcription factors and contain a D-box-binding domain closely related to the PAR proteins *dbp*, *hlf* and *tef*^28^. They play a crucial role in the regulation of light-entrainment of the circadian clock, and thereby, the core clock gene *per2*^32^. Therefore, the results of our present study suggest potential divergent functions of different *nfil3* paralogs in zebrafish, which is consistent with our results observed in the brain^15^ and their differential expression patterns in zebrafish eleuthero-embryos^33^. Moreover, the results support the essential role of the D-box-binding factor, the *nifl3* family, in directing the light-regulated circadian rhythm signals.

Our results indicate that environmental progestins alter the circadian rhythm signals in eyes on the transcriptional level. There is a striking similarity in the reaction in the brain and eyes, although some differences occurred. Both of these organs were responsive to progestins in a dose-dependent manner at the transcriptional level. Several genes displayed relative high transcriptional alterations, such as *nfil3–5* and *nfil3–6*. Overall fold-changes were lower in eyes compared to the brain, and sometimes, did not follow the internal feedback loops. This was possibly due to the lack of normalization in the transcriptional data in our previous study on the brain^15^, as discussed below. Though the underlying molecular mechanisms are still unclear, a recent study revealed that progesterone receptor binding sites exist in the promotor region of several key circadian rhythm genes, such as *clock*, *per1* and *npas2*^34^. Whether these transcriptional alterations translate to physiological endpoints, including alteration in light-response in the eyes or altered locomotor activity should be investigated in forthcoming studies. Furthermore, recent evidence demonstrated that the D-box enhancer would be a general convergence point for light-driven signaling. Light-activated photoreceptor signals can regulate the expressions and functions of the D-box-binding factors, and thereby, directs the light-induced circadian gene containing D-box promoter elements, such as *per* and *cry*^19^. D-box is the binding site for bZIP transcription factors of the proline and acid amino acid-rich (PAR) subfamily (*TEF*, *DBP* and *HLF*), and *E4BP4* (i.e. *NFIL3* family). These transcription factors have been implicated in light-regulated phase shifting of the clock, and in clock output pathways^32^. In the present study, gene expression of these important D-box-binding factors were measured, and some can be activated in response to different concentrations of progestins (*tefb*, *dbpa*, *dbpb*, *nfil3-2* and *nfil3–5*), even at environmental relevant levels (*nfil3-2* and *nfil3–5*). This was consistent with the gene expression changes of several paralogs of *per* and *cry* (Fig. 5). These results support the crucial role of D-box-binding factors in the regulation of light-activated circadian rhythm signals.

### Importance of sampling time

In (eco)toxicological experiments, different exposure levels of chemicals are employed to observe dose-dependency of transcriptional and physiological effects. Due to practical constraints, the time differences between sampling of different groups usually spans up to a few hours. Considering that about 5–15% genome-wide mRNA expressions in vertebrates display significant circadian oscillations during the 24 h period^35^,^36^, a sampling time spanning several hours may result in transcriptional alterations that are not compound-related, but are due to time-related changes regulated by circadian rhythm. Thus, this may result in potential false interpretation of gene expression data.

**Figure 6:**
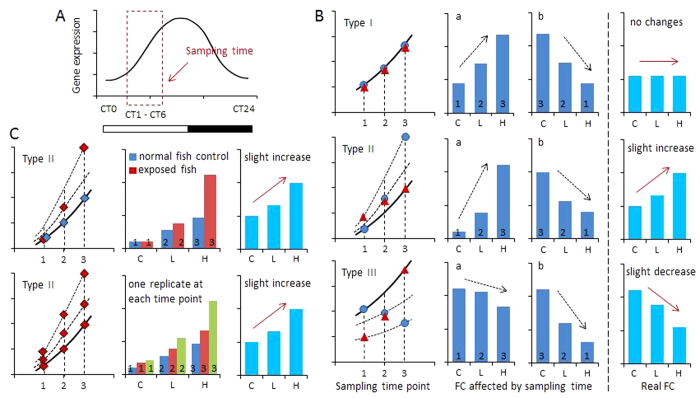
Schematic diagram depicts possible expressional alterations of circadian gene caused by sampling time. (**A**) Representative theoretical example of expressional changes of one circadian gene. Transcriptional level increases at day-time and decreases at night. Sampling time is assumed within a few hours after lights on (CT1–CT6). (**B**) Sampling time affects the interpretation of gene expressions following compound exposure. Three different types (I, II and III) are compared. Type I, no real fold changes (FC); type II, slight increase; type III, slight decrease. Numbers (1, 2 and 3) represent three sampling time points. Three blue dots 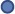 represent sampling order with control (**C**), low dose (L) and high dose (H) of compound (from left to right). Assumed fold-changes are represented in figure a. Three red triangles 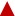 represent reverse sampling order with high dose, low dose and control (from left to right). Assumed fold-changes are represented in figure b. Red solid arrows represent the tendency of assumed real gene expressions; black dashed arrows represent the tendency of gene expressions affected by sampling time. (**C**) Two approaches help to reduce the effects of sampling time, shown for type II expressional changes as example. Upper row: sampling of exposed fish (both solvent control and compound-exposed) and unexposed (normal) fish at each time point. In this case, pairwise comparison can be conducted for each time point. Lower row: one replicate of each exposed group sampled at the same time point with subsequent assessment of average values.

Most circadian rhythm related genes, anticipating the daily light transitions, show peak expressions either immediately before dawn or dusk^36^. To theoretically evaluate the influence of sampling time, we focused on expressional features of circadian rhythm genes, which showed increased expression at day time and decreases at night (Fig. 6A). This pattern is representative to the pattern of *arntl* and *rorc* in zebrafish^21^. In our present study, sampling time occurred within five hours after lights on (CT1–CT6). In general, three different patterns of transcriptional responses are theoretically feasible: no alteration (type I), significant up-regulation (type II) and significant down-regulation (type III). For each pattern, false positive and negative interpretation, respectively, may occur when sampling time of controls and exposed fish did not closely match. This occurred no matter whether mRNA samples were taken in the order of control, low dose and high dose, or reverse (Fig. 6B). For instance, no actual significant alterations occur between controls and treatments in type I pattern, however, when the sampling times differed between controls and treatments, false interpretations may result.

In zebrafish eleuthero-embryos, more than 2800 genes show circadian oscillations during a 24 h period^21^. Among them are circadian rhythm genes, but also widely used biomarker genes in (eco)toxicology, including *ahr1a*, *cyp1a*, *cyp3c4*, *hsd11b2*, *cyp17a1* and androgen receptor *ar*^21^. A similar phenomenon was also observed in other vertebrates^36^, and supposed to exist in adult zebrafish as well. Therefore, care has to be taken to control for correct sampling time and interpretation of transcriptional responses. To avoid false interpretations, one approach is to add unexposed fish as negative controls at each sampling time point as done in our present study. The compound-related transcriptional alterations are detected by comparison of transcriptional levels between exposed fish (both solvent control and compound-exposed) and unexposed fish at each time point. Alternatively, sampling of one replicate of each treatment and control can be performed at the same time point, and subsequently averages and standard deviation can be assessed (Fig. 6C). These two approaches would be accurate in reducing false negative/positive results in experiments.

In conclusion, our transcription data suggest that P4 and DRS can significantly alter the photo-transduction cascade and circadian rhythm on the molecular level in zebrafish eyes. Effects occurred even at environmentally relevant levels (7 ng/L P4). This provides novel insights into this unexpected effect of environmental progestins. Previously, eye dysfunction was reported in fish response to environmental compounds, such as organotins and silver nanoparticles^37^,^38^,^39^,^40^. Whether progestins alter not only gene transcription but translate to behavioral or metabolic responses to light needs to be investigated. This is important considering that circadian rhythm times a variety of crucial cellular and physiological processes in vertebrates, such as energy metabolism, cardiovascular function, sleep-wake rhythm, insulin secretion, hormone secretion and even reproduction^41^.

## Materials and Methods

### Chemicals and maintenance of zebrafish

This study is an extension of our previous study that focused on reproductive effects and circadian rhythm in brain, liver and gonads^15^. Thus, analytical chemical information on exposure levels is given there. Further information can be found in the Supplementary Information (SI).

### Experimental design

Adult female and male zebrafish (10 months old) were exposed for 21 days to a solvent control (0.01% DMSO), and increasing mean measured concentrations of 7, 116 and 742 ng/L P4, and 99, 2´763 and 13´650 ng/L DRS. After a three-day acclimatization, the experiment started with a pre-exposure period of 14 days to establish the baselines in fecundity, followed by one day of equilibration when chemical-dosing started, and 21 days of the progestin exposure period. Each treatment consisted of four replicates and each replicate contained 6 females and 6 males as breeding pairs. The whole experiment was performed by use of a flow-through system, and the parameters for water quality, such as temperature, pH value, conductivity and dissolved oxygen concentration were continuously measured. The photoperiod was 14:10 h light/dark (light on at 8 a.m (CT0) and light off at 10 p.m (CT14)). The study was conducted according to OECD Guideline 229/230, and in accordance with the Swiss Animal welfare regulations. The experimental protocols were approved by the animal welfare authority of the Canton Basel-Stadt, Basel, Switzerland (approval number 2547).

At the end of exposure, fish were anesthetized by KoiMed Sleep (1.5 mL/L water). Eyes of four females and four males per replicate were dissected, and pooled samples (8 eyes per gender per replicate) were transferred to RNAlater and stored at −80 °C for subsequent RNA extraction. Pooling was necessary due to the small tissue sizes, varying extraction efficiencies and to control for inter-individual variability. At the same time, one female and one male fish per replicate (n = 4 per gender per treatment) were fixed in Bouin’s solution for histological examination. Due to the practical constraints, time differences between sampling of solvent controls and progestin exposed zebrafish spanned up to 5 hours (CT1–CT6). Control fish were first sampled at 9 a.m (CT1), followed by fish treated with P4 and DRS (each from low to high doses) with an approximate interval of 50 min for each treatment.

In order to perform a time-specific comparison, a second sampling was performed employing unexposed zebrafish with the same strain, age and breeding conditions. Sampling of tissues was performed at different time points spanning the whole sampling period that was used in the progestin-exposure experiment. Thus, 8 eyes/gender/replicate/time-point were sampled. These samples were used as a negative control.

### Histology

Histological analysis of zebrafish eyes was performed according to the protocols described previously^14^,^15^. In brief, four males and four females per treatment (one male and one female per biological replicate) were randomly taken after anesthesia and fixed in Bouin’s solution for about 24 h. After fixation, fish were kept in 70% ethanol for about 4–6 weeks. Slices were then prepared and histological analyses were conducted by standard hematoxylin −eosin (HE) staining.

### RNA Isolation and qRT-PCR analysis

Total RNA was extracted from pooled zebrafish eyes (both for exposed fish and unexposed fish, 8 eyes per gender per biological replicate) by use of the RNeasy Mini Kit (Qiagen, Basel, Switzerland). The samples were then purified and DNase and divalent cations were removed by use of RNase-free DNase (Qiagen, Basel, Switzerland). RNA concentrations were analyzed by a NanoDrop 1000 spectrophotometer (Nanodrop Technologies Inc. Wilmington DE, U.S.) by measuring the absorbance at 260 and 280 nm; the purity of each sample was between 1.8 and 2.0 (260/280 nm ratio). RNA samples were then stored at −80 °C for subsequent RT-qPCR analysis.

The first-strand cDNA synthesis and real time RT-PCR was performed according to methods described previously^11^,^14^. Briefly, RNA samples were reverse-transcribed by Moloney murine leukemia virus reverse transcriptase (MMLV) in the presence of random hexamers and deoxynucleoside triphosphate (dNTP). RT-qPCR was conducted on Biorad CFX96 real time PCR detection system based on SYBR green fluorescence. Of 46 pairs of primers used, 14 pairs were designed in the present study and other 32 pairs were obtained from the published zebrafish primer sequences (Table S1).

For primer design, Primer-BLAST (http://www.ncbi.nlm.nih.gov/tools/primer-blast/) was employed, and the primers spanning the intron/exon boundary were preferenced to minimize DNA contamination. Melting curves were analyzed for all these genes to ensure that only a single product was amplified, and the efficiencies were calculated (90–110% for all the primers) to ensure that no significant changes between the primer efficiencies of target genes and the reference gene, *β-actin*. *β-actin* was selected as housekeeping gene for normalization, because it displayed higher stability in expression at different treatments and time points compared to other reference genes, *rpL13a* and *18s* (Fig. S3). For calculating the expression levels, threshold cycle (CT) values were recorded in the linear phase of amplification and delta−delta CT method of relative quantification was used, as previously described^14^.

### Statistical analysis

A hierarchical clustering map was constructed with MultiExperimental Viewer v4.9 (Dana-Farber Cancer Institute, Boston, MA). Data of gene expressions for eyes was graphically illustrated and statistically analyzed by GraphPad Prism 5 (GraphPad Software, San Diego, CA, USA). The significant differences between treatments at each time point were assessed by one-way analysis of variance (ANOVA) to compare with the normal zebrafish sampled at the same time point, followed by a post-hoc Tukey’s test. Before running the ANOVA, data was tested for the homogeneity of variances using Levene’s test. The data were log-transformed, where required. Results are given as mean ± standard deviation (S.D.). Significant differences were considered when p ≤ 0.05.

## Additional Information

**How to cite this article**: Zhao, Y. and Fent, K. Progestins alter photo-transduction cascade and circadian rhythm network in eyes of zebrafish (*Danio rerio*). *Sci. Rep.* **6**, 21559; doi: 10.1038/srep21559 (2016).

## Supplementary Material

Supplementary Information
