# Supplementary material for: Progestins alter photo-transduction cascade and circadian rhythm network in eyes of zebrafish (Danio rerio)
Source: Sci Rep. 2016 Feb 22;6:21559. doi: 10.1038/srep21559 (PMC4761927; doi:10.1038/srep21559)
Supplement: Supplementary Information [file srep21559-s1.doc]

**Supplementary information**

**Progestins alter photo-transduction cascade and circadian rhythm network in eyes of zebrafish (*Danio rerio*)**

Yanbin Zhao^1^, and Karl Fent^1,2^,*

^1^ University of Applied Sciences and Arts Northwestern Switzerland, School of Life Sciences, Gründenstrasse 40, CH–4132 Muttenz, Switzerland

^2^ Swiss Federal Institute of Technology (ETH Zürich), Institute of Biogeochemistry and Pollution Dynamics, Department of Environmental System Sciences, CH–8092 Zürich, Switzerland

* **Corresponding author:**

Prof. Dr. Karl Fent

Tel.: +41 61 467 4571; Fax: +41 61 467 47 84

E-mail: [karl.fent@fhnw.ch](mailto:karl.fent@fhnw.ch); [karl.fent@bluewin.ch](mailto:karl.fent@bluewin.ch%0C)

Number of pages: 9

Number of figures: 3

Number of tables: 1

**Materials and methods**

***Chemicals.*** Progesterone (P4) (purity ≥ 99%), drospirenone (DRS) (purity ≥ 98%) and dimethylsulfoxide (DMSO) (purity ≥ 99.5%) were purchased from Sigma-Aldrich (Fluka AG, Buchs, Switzerland). KoiMed Sleep (Ethylene glycol monophenyl ether) was purchased from KOI&BONSAI Zimmermann (Bühlertann, Switzerland). Bouin’s solution (HT10132) used for fixation was obtained from Sigma-Aldrich (Fluka AG, Buchs, Switzerland). The kits used for RNA extraction (RNeasyMini Kit 74104 and RNase-Free DNase Set 79254) and enzymes for cDNA synthesis (M-MLV Reverse Transcriptase (M1705)) were purchased from Qiagen (Basel, Switzerland) and Promega (Dübendorf, Switzerland), respectively. SYBR Green fluorescence for real time RT-PCR was obtained from Roche Diagnostics (Roche Diagnostics, Switzerland).

***Maintenance of Zebrafish.*** Zebrafish care and maintenance was performed as described previously^1^. In brief, adult zebrafish (*Danio rerio*, 3 months old) were obtained from Harlan Laboratories, Inc. (Itingen, Switzerland), and transferred into 300 L culture tanks (with 240 L fish water) for acclimatization for more than three months. Fish were held in reconstituted deionized water (salts: CaCl_2_×2H_2_O 147.0 g/L, KCl 2.9 g/L, MgSO_4_×7H_2_O 61.6 g/L, NaHCO_3_ 32.4 g/L) with a conductivity of 470–480 μS/cm. Water was renewed each week and held constant at 27±1 °C. The photoperiod was 14:10 h light/dark. Fish were fed twice daily with a combination of frozen brine shrimps (*A. salina*), white mosquito larvae and *Daphnia magna*. Water parameters, such as nitrate, nitrite and pH, were controlled regularly using Test strips (Easy Test, JBL) and the oxygen concentration was always ≥80%.

**Table S1.** Primer sequences for quantitative real-time PCR analysis and sources. For the primers designed in our lab, intron/exon boundary-spanning primers were preference to minimize the genomic DNA contamination. Primer efficiency values are ranging from 90% to 110%.

| Gene | Gene Bank no. | Sense primer (5’-3’) | Antisense primer (5’-3’) | Product size (bp) | |
| --- | --- | --- | --- | --- | --- |
| *rpl13α*^a^ | NM_212784 | AGCTCAAGATGGCAACACAG | AAGTTCTTCTCGTCCTCC | | 100 |
| *β-actin*^b^ | AF057040.1 | TCTGGCATCACACCTTCTACAAT | TGTTGGCTTTGGGATTCAGG | | 97 |
| *18s*^c^ | BX296557 | CCACTCCCGAGATCCAACTA | CAAATTACCCATTCCCGACA | | 215 |
| *opn1sw2* | NM_131192.1 | ATCTGGGTGGTTTCCAACCG | ACAGGAGCGGAACTGTTTGTT | | 132 |
| *opn1mw2* | NM_182891.2 | TCTCACGTCTCTCCACCGAT | GCTGAACCCTTGGGTGAGTT | | 133 |
| *rho*^d^ | [NM_131084.1](http://www.ncbi.nlm.nih.gov/entrez/viewer.fcgi?db=nucleotide&id=18859316) | ACTTCCGTTTCGGGGAGAAC | GAAGGACTCGTTGTTGACAC | | 176 |
| *gnat1*^d^ | [NM_131868.2](http://www.ncbi.nlm.nih.gov/entrez/viewer.fcgi?db=nucleotide&id=41282134) | CCGTTACTTCGCCACCACAT | GAAGGTGTTGGGACCGTCAT | | 121 |
| *gnb1a*^d^ | [NM_212609.1](http://www.ncbi.nlm.nih.gov/entrez/viewer.fcgi?db=nucleotide&id=47086810) | TGCCACCCTCTCTCAGATCA | CAAGTGTCCCCTCAGTGTCC | | 85 |
| *pde6a*^d^ | [NM_001007160.2](http://www.ncbi.nlm.nih.gov/entrez/viewer.fcgi?db=nucleotide&id=360039205) | CAGTCAACAAGATCGGGGCT | GCTCAGGTGAAACACTCGGA | | 104 |
| *cnga1a*^d^ | [XM_695944.7](http://www.ncbi.nlm.nih.gov/entrez/viewer.fcgi?db=nucleotide&id=688612871) | ATCGCAGAACCGCCAACATA | CGGATATTCAGTCAGCGCCT | | 92 |
| *grk1a*^d^ | [NM_001034181.2](http://www.ncbi.nlm.nih.gov/entrez/viewer.fcgi?db=nucleotide&id=99028948) | GCTGTACGCCTGCAAGAAAC | TCCACCATCGCTCCCTCATA | | 72 |
| *arr3a*^d^ | NM_001002405.1 | GAGGAGAAGATCGCCCATCG | CTGGCCATTTCCACTGGTCT | | 93 |
| *gucy2f* | [NM_131864.2](http://www.ncbi.nlm.nih.gov/nucleotide/169259779?report=gbwithparts) | GGATCGATCGCGCTCTGTAA | CGCAGGTCCTTCATCTTGGT | | 165 |
| *clock1*^e^ | XM_009294633.1 | GGTTCAAGGACAGGGTTTACAGATG | GGTCGA CCTCTGAGACTGCTGG | | 280 |
| *clock3*^e^ | XM_005168339.2 | GAGAGTACAGGGACCTCAGATGATC | ATACACAGGACCGCACTGAGTTAC | | 268 |
| *arntl1a*^e^ | XM_009297921.1 | GTCACAGACAAGTGCTACAGATGCG | TCCCTCCGCCATCTCCTGA | | 261 |
| *arntl1b*^e^ | XM_009303573.1 | TGACGGCTCAGGGAA AACC | GAGAATTGTCACTTAAAATGGAGCTG | | 305 |
| *arntl2*^a^ | XM_005169955.2 | GTGTCAACCAACACGGTTGTATCC | TGGAACTTGTTGGGATTTCTTGGC | | 120 |
| *per1a*^a^ | [XM_005172626.2](http://www.ncbi.nlm.nih.gov/entrez/viewer.fcgi?db=nucleotide&id=688574024) | ATGCGTGCAAGAAGTGGTG | ACGTCCTCATTTAGCGGACTC | | 131 |
| *per1b*^e^ | NM_212439 | CCTCCTGAGTCAGATATCGTAATGG | GCAGCGCACACCTCTTGATAA | | 324 |
| *per2*^e^ | [XM_009298837.1](http://www.ncbi.nlm.nih.gov/entrez/viewer.fcgi?db=nucleotide&id=688539158) | GTGGAGAAAGCGGGCAGC | GCTCTTGTTGCTGCTTTCAGTTCT | | 252 |
| *per3*^e^ | [NM_131584.1](http://www.ncbi.nlm.nih.gov/entrez/viewer.fcgi?db=nucleotide&id=18859228) | CCACAGCCTGAGTCCGAAGTC | CCCCTCTGTGATGTGAATGTGC | | 286 |
| *cry1a*^e^ | NM_001077297.2 | CTACAGGAAGGTCAAAAAGAACAGC | CTCCTCGAACACCTTCATGCC | | 334 |
| *cry1b*^e^ | [NM_131790.4](http://www.ncbi.nlm.nih.gov/entrez/viewer.fcgi?db=nucleotide&id=148540004) | CTACAGGAAGGTAAAGAAGAACAGCA | CAACAACTCCTCAAACACCTTCAT | | 340 |
| *cry2a*^e^ | [XM_005166893.2](http://www.ncbi.nlm.nih.gov/entrez/viewer.fcgi?db=nucleotide&id=688567517) | GGACCAATACACCAGCACCAG | CAGCAAGTGTCCTGCCATGTC | | 245 |
| *cry2b*^e^ | [XM_005168280.2](http://www.ncbi.nlm.nih.gov/entrez/viewer.fcgi?db=nucleotide&id=688607062) | ATCGTCTTATACAGGGGTCAGGAG | CTTCCCGCCTCTCGTTGTC | | 287 |
| *cry3* | [NM_131786.2](http://www.ncbi.nlm.nih.gov/entrez/viewer.fcgi?db=nucleotide&id=40254687) | TGCATTGGTTTCGCAAAGGG | AGACATCTGTTGGCTGTCCG | | 234 |
| *cry4* | [NM_131787.1](http://www.ncbi.nlm.nih.gov/entrez/viewer.fcgi?db=nucleotide&id=18858470) | AGGAGGGCATGAAGGTGTTC | CACCGGACAGAAGATCCTGG | | 125 |
| *cry-dash* | [NM_205686.1](http://www.ncbi.nlm.nih.gov/entrez/viewer.fcgi?db=nucleotide&id=45387782) | CAGAGCCATTGGACGACTGT | TGCGAGCCAAGGAGAAAACT | | 173 |
| *cry5*^a^ | NM_131788.1 | CATGGAGAGAACGAACTGGG | GTGCAGACAAGCAGCCGAAC | | 116 |
| *nr1d1*^a^ | NM_205729 | GTGAACAACCAGCTGCAGAA | ACTGTAAGGCCTGGACATGG | | 125 |
| *nr1d2a*^e^ | NM_001130592.1 | CATGTCAAGAGACGCCGTGC | GGGACAAACCAGATGTGCTCG | | 478 |
| *nr1d2b*^e^ | NM_131065 | GCACCTGGTCTGCCCGA | CGGACCACCAGCACCTCA | | 207 |
| *rorca* | NM_001082819.1 | TCTGTCTCCAGACCGACCTT | CTCCTCATGGACACCGCTTT | | 112 |
| *rorcb* | NM_001277094.2 | AAGCAGGATGCCTGGAAGTC | GTCATCACACCCGAGAGCTT | | 125 |
| *ciart* | [XM_001923066.4](http://www.ncbi.nlm.nih.gov/entrez/viewer.fcgi?db=nucleotide&id=688599086) | GCGTGCGATCAAGACAATCC | TCGGTACCACAACAACCTCG | | 116 |
| *dec-1*^f^ | [NM_212679.2](http://www.ncbi.nlm.nih.gov/entrez/viewer.fcgi?db=nucleotide&id=114703729) | AAGGCTCTTAACAACCTGCTG | GAACGAAACATCTCTTCACTGTT | | 119 |
| *dec-2*^f^ | [NM_001039107.1](http://www.ncbi.nlm.nih.gov/entrez/viewer.fcgi?db=nucleotide&id=84993731) | ACGGGGAGCGATCGCTGAAG | TGGTTGATGAGTCGCGTGCAC | | 151 |
| tefa^g^ | [XM_005156135.2](http://www.ncbi.nlm.nih.gov/entrez/viewer.fcgi?db=nucleotide&id=688579631) | AAGGCAATAAATGAATAATAGTTTGGA | TCACCTGCTTCTATCTTGTCTCC | | 60 |
| *tefb*^g^ | [XM_005156135.2](http://www.ncbi.nlm.nih.gov/entrez/viewer.fcgi?db=nucleotide&id=688579631) | GTGCCTGAGGATCAGAAGGA | GATCGTTTGGCTGCAACAT | | 71 |
| *dbpa* | [NM_001197060.1](http://www.ncbi.nlm.nih.gov/entrez/viewer.fcgi?db=nucleotide&id=308153227) | GCTCCCAATGTCCACCTTCA | CCATCACATCAGCCGCATTG | | 240 |
| *dbpb* | [XM_005157897.2](http://www.ncbi.nlm.nih.gov/entrez/viewer.fcgi?db=nucleotide&id=688590070) | ACAGACTGCTTGCATGGCAC | TGATGTCGGCGTGGATCAAA | | 194 |
| *nfil3*^h^ | [NM_001004120.2](http://www.ncbi.nlm.nih.gov/entrez/viewer.fcgi?db=nucleotide&id=402747141) | GGTTACTAGAGATAACCACTGAATTC | CATCTCATCGTAGACCAAGTGC | | 90 |
| *nfil3-2* | [NM_001197065.1](http://www.ncbi.nlm.nih.gov/entrez/viewer.fcgi?db=nucleotide&id=308153231) | AGTCAAAGGCACTGCCCTAC | GCGTCATGGGTGAGTCTTGA | | 92 |
| *nfil3-5* | [XM_005165724.2](http://www.ncbi.nlm.nih.gov/entrez/viewer.fcgi?db=nucleotide&id=688558727) | CTTCCAACCCAAAACAGCGG | GCAGCCTCGTTATTCTTGCG | | 218 |
| *nfil3-6* | [NM_001002218.1](http://www.ncbi.nlm.nih.gov/entrez/viewer.fcgi?db=nucleotide&id=50345093) | TTTGCTTGCACGCTCACTTC | TTACACGGCGTTTCTCACGA | | 188 |

Data sources: *a(1); b(2); c(3); d(4); e(5); f(6); g(7); h****(****8)*

**Figure S1.**

Relationships between the transcriptional levels (represent as CT values) of unexposed (normal) zebrafish eyes sampling at 8:30 a.m. (0.5h after light on) and DMSO exposed zebrafish eyes sampling at the same time point.


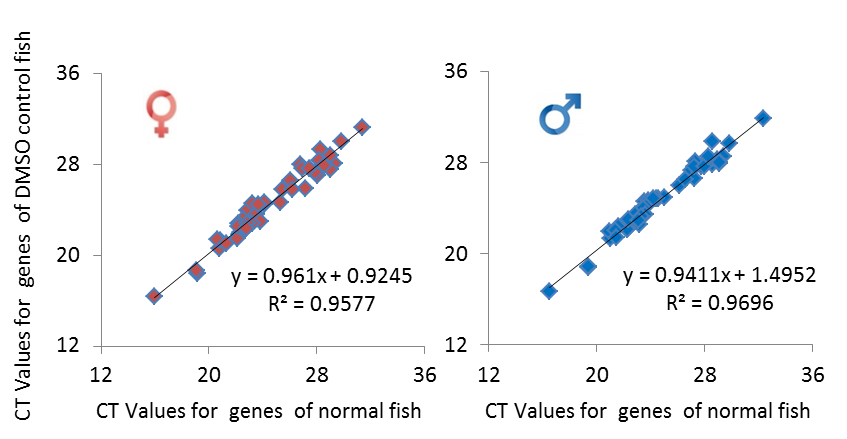


**Figure S2.**

Transcriptional responses of all photo-transduction genes in zebrafish eyes. Genes expressed as the fold-changes compared to unexposed (normal) fish sampled at the same time-point. Blue bars in each figure represent gene expressions of unexposed (normal) fish. Red bars in each figure represent gene expressions of exposed fish; C, solvent control and P4 and DRS-exposed. Key for concentrations (red bars): P4: L: low dose (7 ng/L); M: middle dose (116 ng/L); H: high dose (742 ng/L). DRS: L: low dose (99 ng/L); M: middle dose
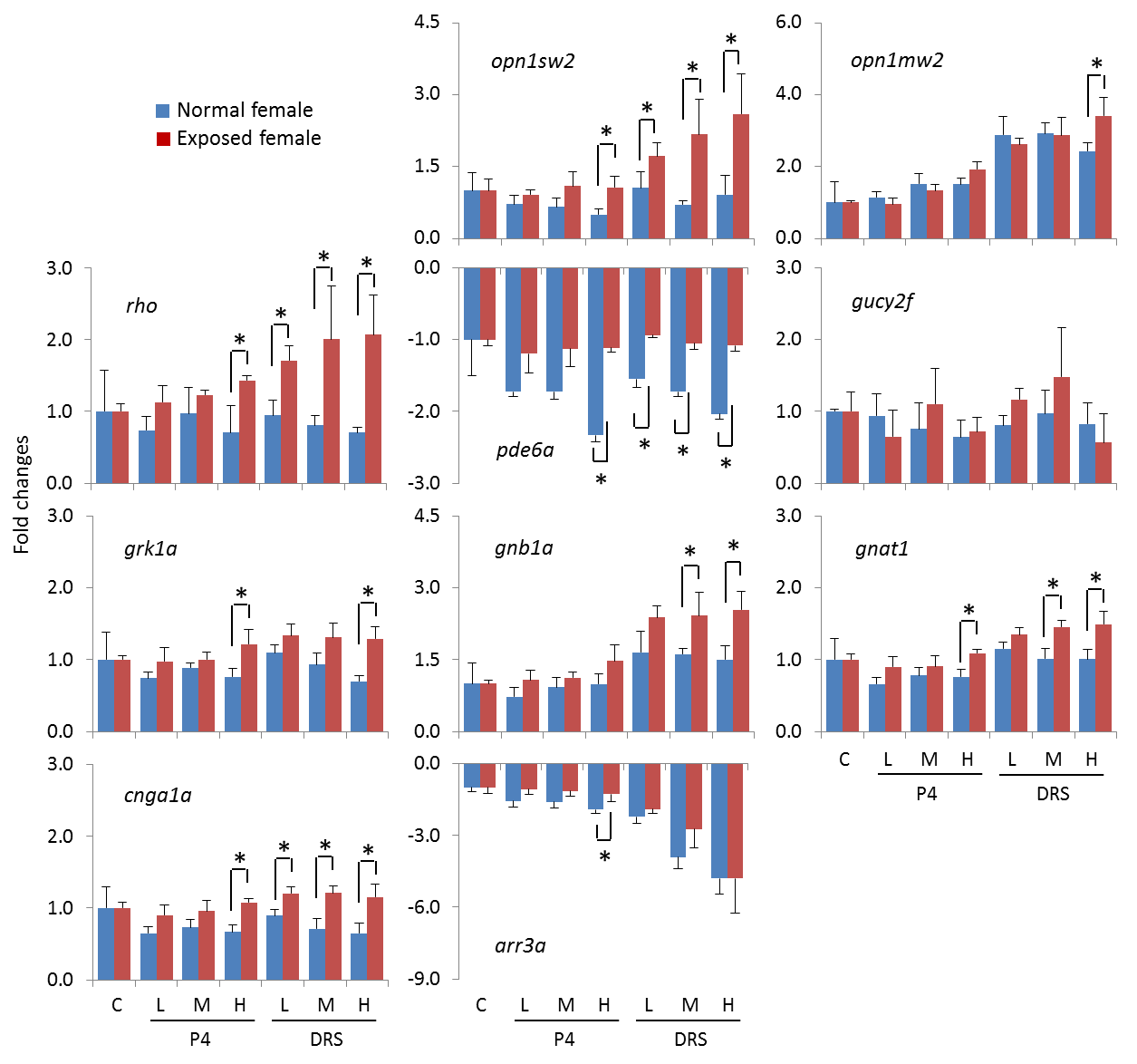
(2´763 ng/L); H: high dose (13´650 ng/L).


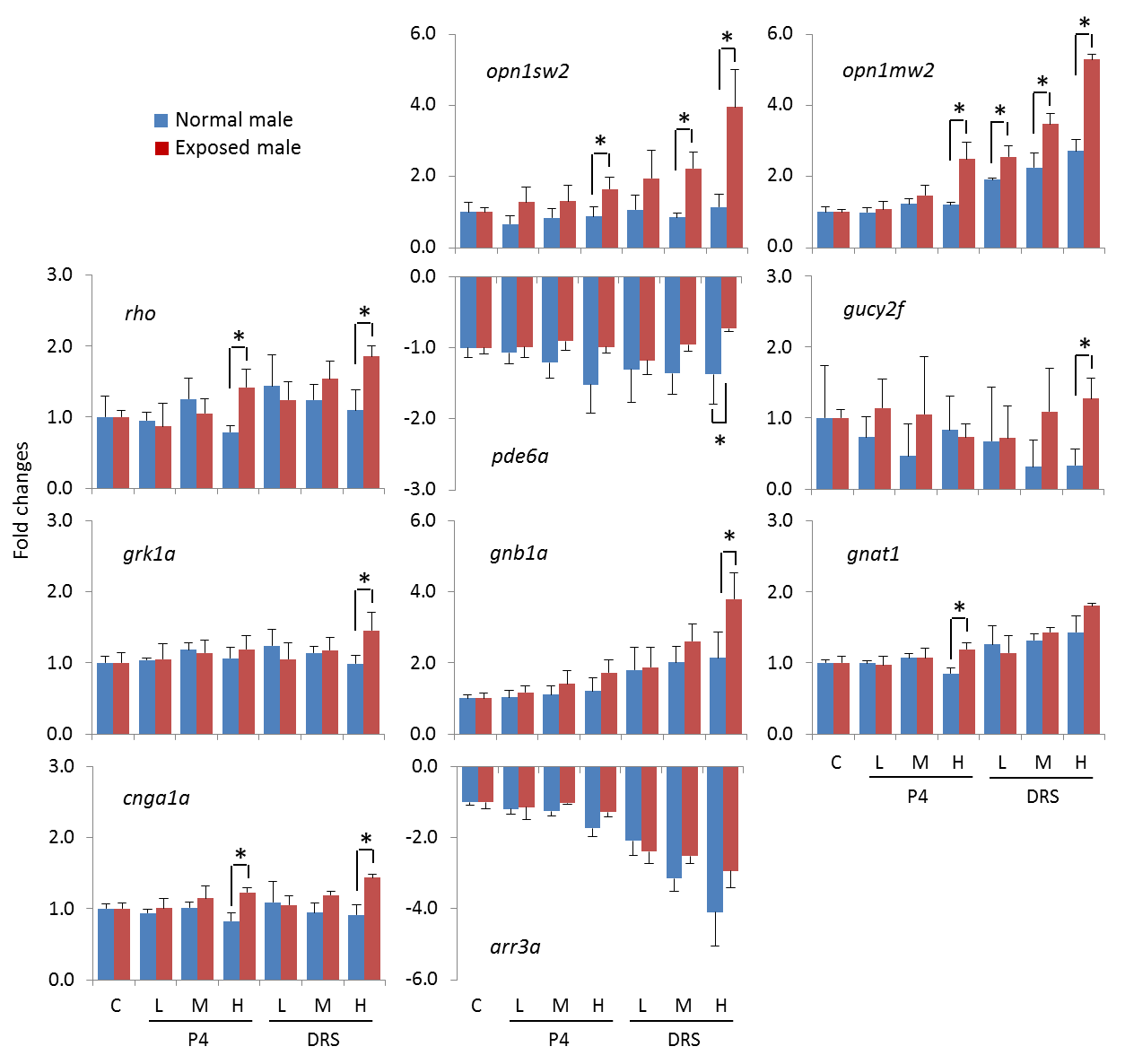


**Figure S3**.

Stability analysis of three housekeeping gene expressions (*rpl13a*, *b-actin* and *18s*) in eyes of zebrafish exposed to solvent control, and different concentrations of P4 and DRS. Each bar represents the mean value ± S.D. of 4 replicates (for each replicate: four fish/8 eyes pooled) per group.


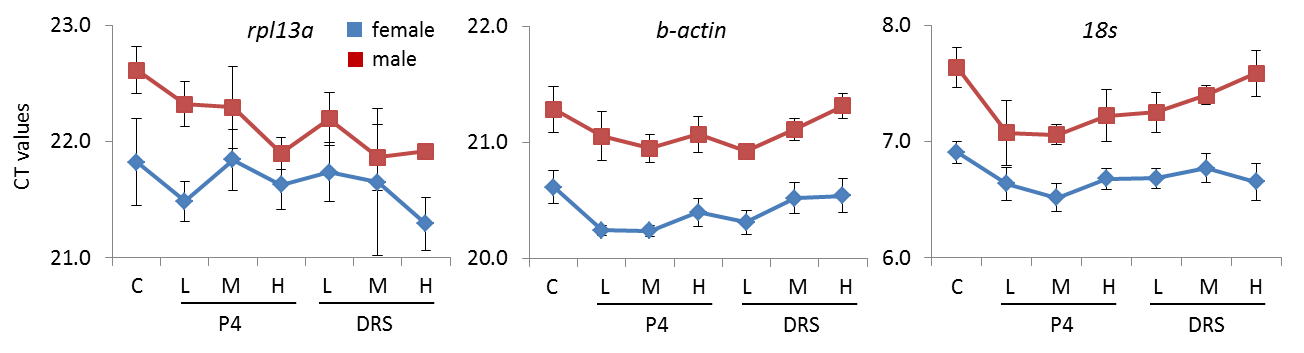


***References***

1. Zucchi, S., Mirbahai, L., Castiglioni, S. & Fent, K. Transcriptional and physiological responses induced by binary mixtures of drospirenone and progesterone in zebrafish (*Danio rerio*) at environmental concentrations. *Environ. Sci. Technol.* **48,** 3523–3531 (2014).
2. Liang, Y. Q. *et al.* Progesterone and norgestrel alter transcriptional expression of genes along the hypothalamic–pituitary– thyroid axis in zebraﬁsh embryos-larvae. *Comp. Biochem. Physiol. Part C: Toxicol. Pharmacol*. **167,** 101–107 (2015).
3. Meng, X., Bartholomew, C. & Craft, J. A. Differential expression of vitellogenin and oestrogen receptor genes in the liver of zebrafish (*Danio rerio*). *Anal. Bioanal. Chem.* **396,** 625–630 (2010).
4. Laranjeiro, R. & Whitmore, D. Transcription factors involved in retinogenesis are co-opted by the circadian clock following photoreceptor differentiation. *Development* **141,** 2644-2656 (2014).
5. Oggier, D. M., Weisbrod, C. J., Stoller, A. M., Zenker, A. K. & Fent, K. Effects of diazepam on gene expression and link to physiological effects in different life stages in zebrafish *Danio rerio*. *Environ. Sci. Technol.* **44,** 7685-7691 (2010).
6. Amaral, I. P. G. & Johnston, I. A. Circadian expression of clock and putative clock-controlled genes in skeletal muscle of the zebrafish. *Am. J. Physiol. Regul. Integr. Comp. Physiol.* **302,** R193–R206 (2012).
7. Abe, T. *et al.* Molecular analysis of Dec1 and Dec2 in the peripheral circadian clock of zebrafish photosensitive cells. *Biochem. Biophys. Res. Commun.* **351,** 1072–1077 (2006).
8. Gavriouchkina, D. *et al.* Thyrotroph embryonic factor regulates light-induced transcription of repair genes in zebrafish embryonic cells. *PLoS ONE* **5,** e12542 (2010).
